# Neuroendocrine Carcinoma Presenting as Metastasis of Unknown Origin in Cervical Lymph Nodes: An Unwonted Case Report

**DOI:** 10.1007/s12070-025-05425-4

**Published:** 2025-04-28

**Authors:** Anmol Kath, Prince Handa, Aparna Ganesan, Nishtha Batra

**Affiliations:** 1 Head & Neck Oncology, Homi Bhabha Cancer Hospital & Research Center, Sangrur, India; 2 Dept of Onco-Pathology, Homi Bhabha Cancer Hospital & Research Center, Sangrur, India

**Keywords:** Neuroendocrine carcinoma, Metastasis of unknown primary, Neo-adjuvant chemotherapy

## Abstract

Neuroendocrine carcinomas occurring in cervical lymph nodes are few and far between. Such presentations demand meticulous clinic-radiological workup besides appropriate immunohistochemical evaluation. We present an adequately managed case of cervical lymph nodal metastatic carcinoma with neuroendocrine differentiation.

## Introduction

Neuroendocrine carcinomas (NECs) of the head and neck region are thin on the ground. The diagnosis of such tumors remains intricate due to the indispensable need for specific immunohistochemical (IHC) analyses for confirmation [1]. The existing literature reports a meager incidence of NECs in the head and neck region, with most tumors epicentered over the sinonasal region and larynx. We present a case of cervical lymph node metastasis from an unknown origin (MUO) which was histologically diagnosed as metastatic carcinoma with neuroendocrine differentiation.

## Case Report

A female patient in her early 70s presented to head and neck oncology OPD with a 4-month history of a painless, progressive swelling over the right side of neck, which was not associated with any other symptoms such as dyspnea, dysphagia, voice change, cough, fever, night sweats, and loss of weight or appetite. The medical and family history was non-contributory. Clinical examination revealed presence of a well-defined hard and fixed right level II nodal mass, measuring approximately 6 cm x 5 cm. It was non-tender, non-pulsatile, with no skin fixity. Fiberoptic laryngoscopy (FOL), fiberoptic bronchoscopy (FOB) and upper gastrointestinal endoscopy (UGIE) did not reveal any significant lesion. A whole-body PET CT highlighted an FDG avid, enhancing, nodal lesion involving the right cervical level II and III stations, measuring 6.1 cm x 5.2 cm with SUV max of 31. The right IJV was not appreciable at the level of the lesion. The angle of contact with right CCA was 180 degrees and with proximal ICA was 90 degrees. The scan confirmed no other FDG avid lesion elsewhere in the body. Ultrasound guided core biopsy from the nodal mass was comprised of large, pleomorphic, tumor cells with conspicuous eosinophilic nucleoli, suggestive of a poorly differentiated malignancy. On IHC, the tumor cells were positive for AE1/AE3, EMA and INSM1 while negative for CD30, CD 3, CD 20, PAX 5, CK5/6, LCA, synaptophysin, CK7, CK20, p63, CD56, S-100, MUM 1, p40 and Melan-A. INI 1was retained. Ki-67 is 88–90% in the highest proliferating area, hence ascertaining the diagnosis as Metastatic carcinoma with Neuroendocrine differentiation. After speculation in the multidisciplinary meeting, the patient was planned for neoadjuvant chemotherapy (NACT) followed by surgery. The NACT regimen comprised of 3 cycles of Etoposide and Carboplatin, which was administered over 6 weeks. The response assessment PET-CT demonstrated non-FDG avid soft tissue density lesion with reduction in size to 1.4 × 1.8 cm, and maintained fat planes with the great vessels, indicating a favorable response. The patient then underwent type III MND on the right neck under general anesthesia. The post-operative phase was uneventful. Based on the post–operative histopathological findings of one metastatic lymph node with neuroendocrine differentiation, the patient was planned for adjuvant chemoradiation.

## Discussion

MUO presentation in the neck accounts for about 5–10% of all carcinomas of unknown primary (CUP) [2]. Metastases noted in the upper neck sites are usually squamous cell carcinomas by histology, whereas those in the supraclavicular region often correlate with primaries of the thoracic region or lymphomas [2]. Interestingly, in this case report the MUO was diagnosed to be metastatic carcinoma of neuroendocrine differentiation.

The pioneer report on NEC in lymph nodes was by Eusebi et al. [3] in 1992. They described neuroendocrine differentiation in 8 cases of CUP, of which one was in the submandibular region. The authors advocated that the occurrence of these tumors could be attributed to anomalous differentiation of mesenchymal stem cells of the lymphoreticular system [3]. More recently Bouzbouz et al. [1] in 2020 described a small cell neuroendocrine carcinoma manifesting as a cervical mass, which was managed with 2 cycles of decompressive radiation therapy only.

Van der Laan et al. [4] in their meta-analysis of 436 cases of NEC in larynx have proposed treatment recommendations ranging from local surgical excision, radiation, chemotherapy or a combination. Prognostically, these tumors have an overall survival rate of 33%- and 5-year DFS of 25%. Local and distant failures at 5 years were 23% and 71% respectively [4]. However, until date there has been no consensus or guidelines pertaining to the treatment of NECs of the head and neck, with multimodality treatment being advocated in majority of the cases. The beneficial role of chemotherapy in management of NECs has been highlighted in the literature [5]. Commonly used regimen was Cisplatin/Carboplatin with Etoposide [5, 6].

**Fig. 1:**
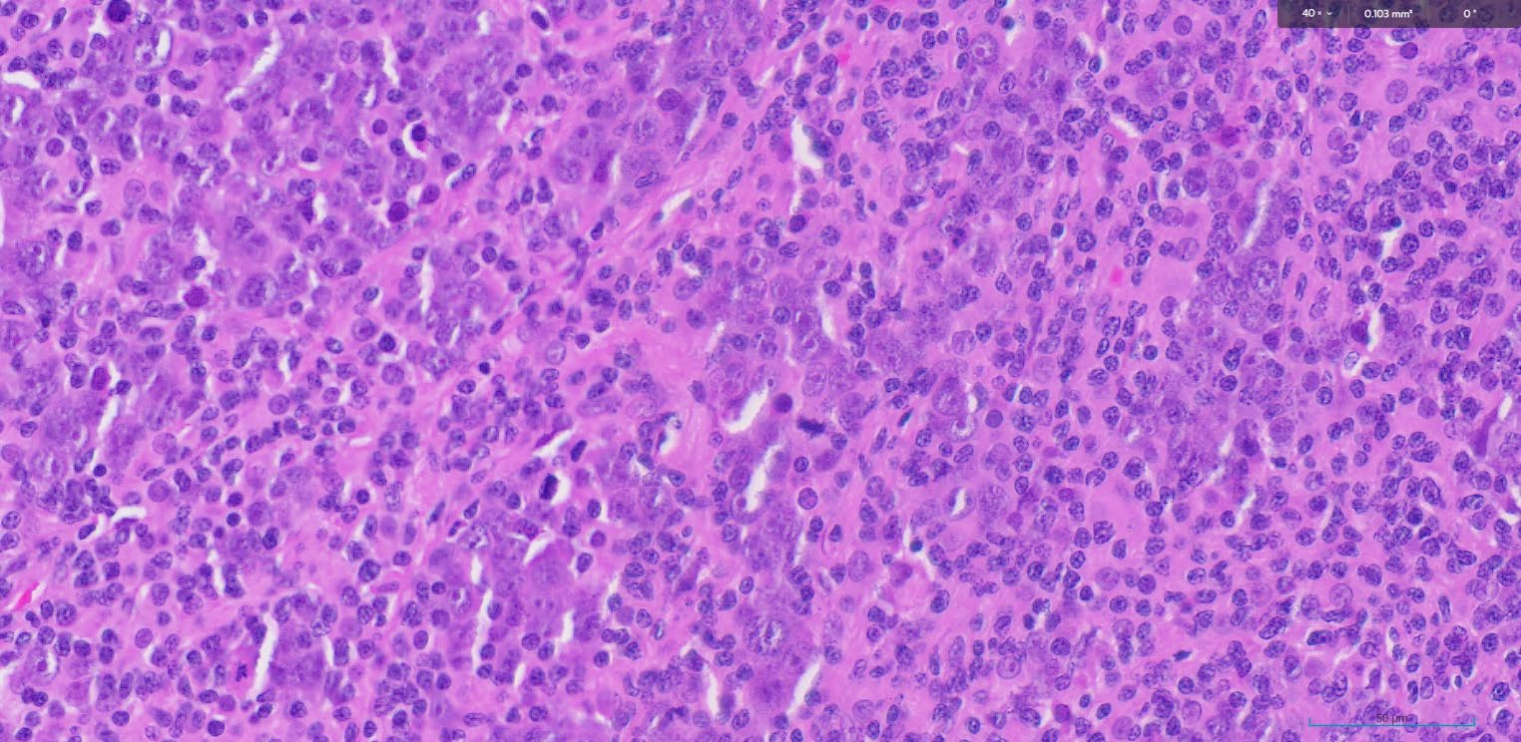
Focus with metastatic deposit (Hematoxylin & Eosin, 400x)

**Fig. 2:**
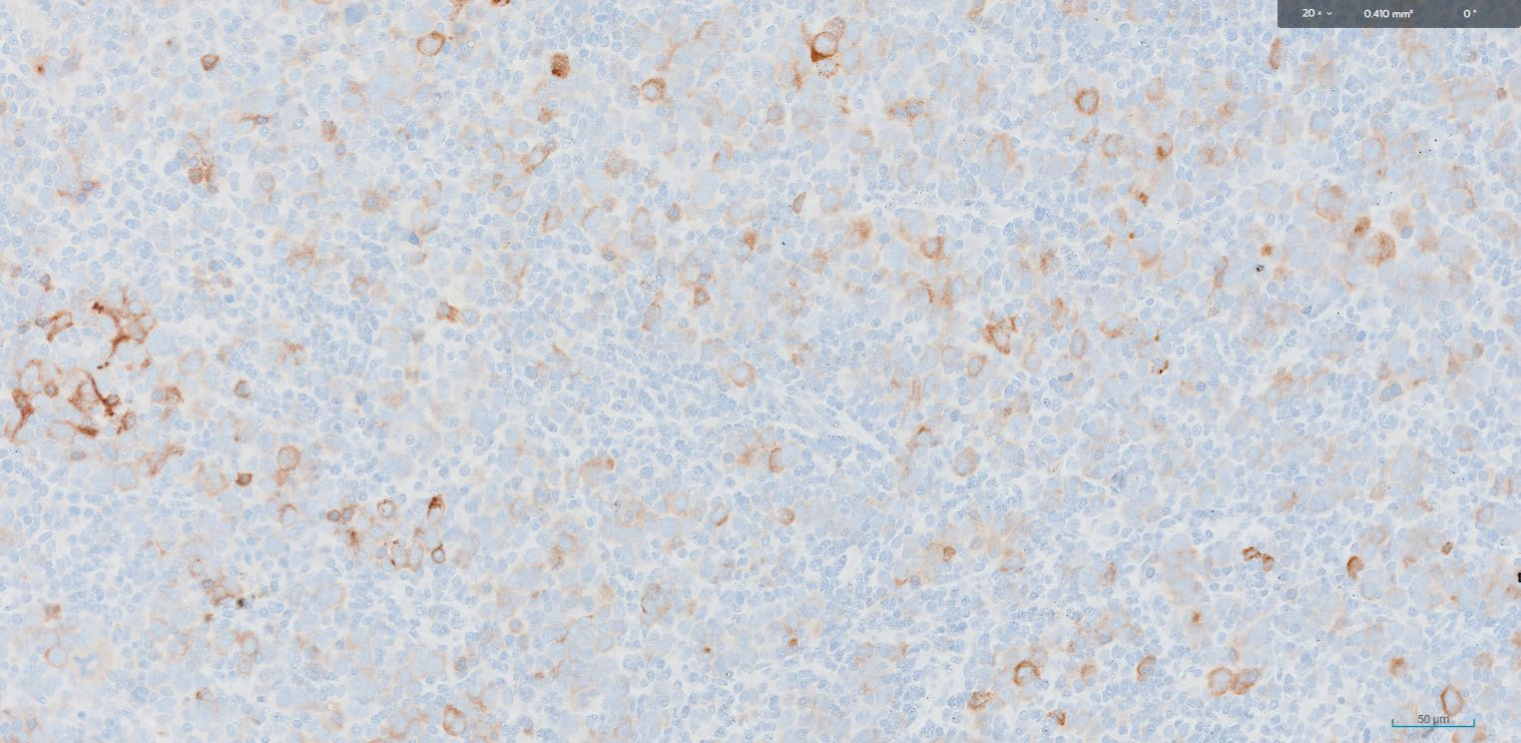
Immunohistochemistry for EMA, positive in tumour cells (400x)

**Fig. 3:**
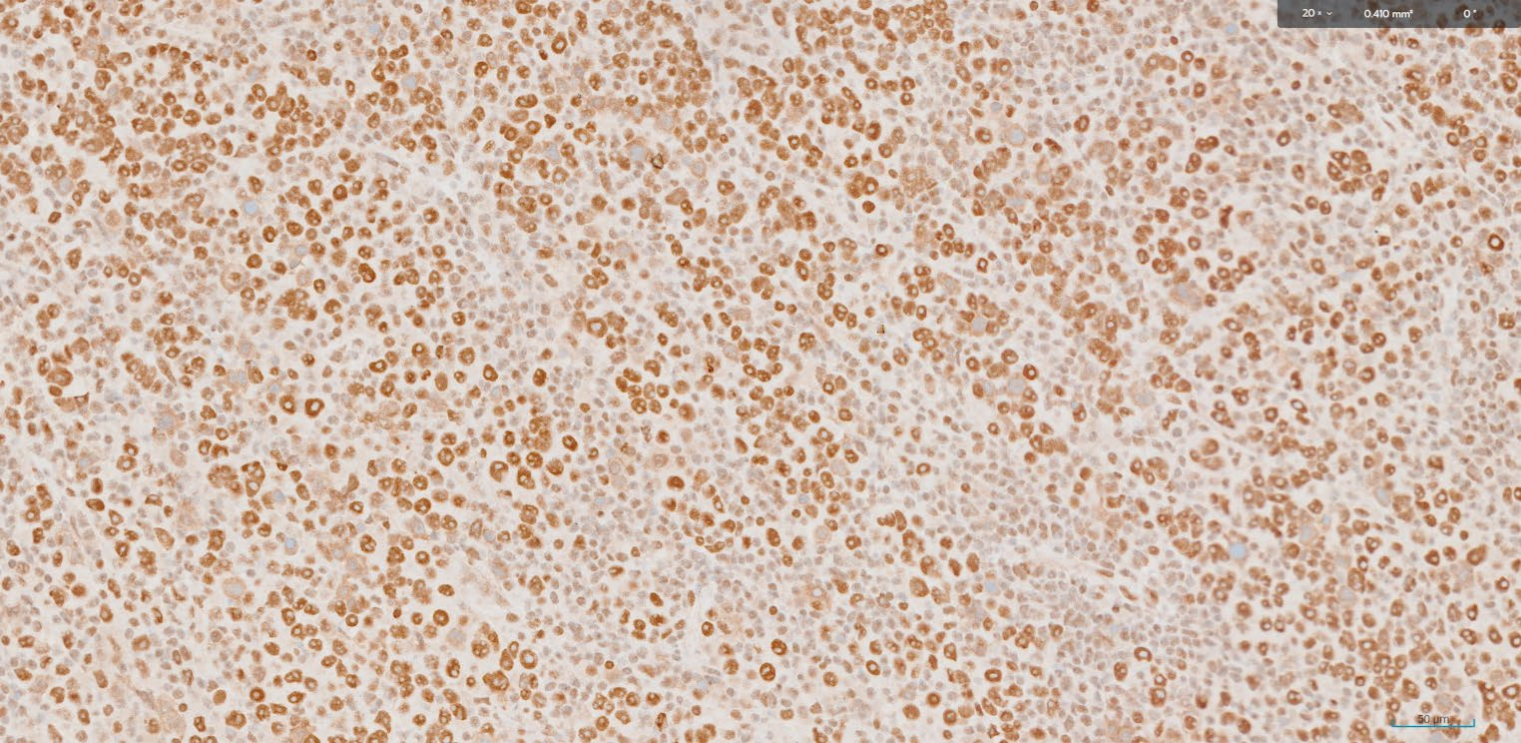
Immunohistochemistry for INI 1, tumour cells show retained expression (400x)

In the present case, a clinical diagnosis of MUO neck was ascertained as triple endoscopy and whole-body PET -CT demonstrated no other primary pathology. Histopathological diagnosis of Metastatic carcinoma of neuroendocrine differentiation was endorsed by immunomorphological evaluation of core biopsy. A multidisciplinary committee speculated the treatment plan as NACT followed by reassessment for surgical resectability. The decision on NACT was made due to the following three reasons. Firstly, there was 180degree CCA encasement which rendered the tumor borderline unresectable by upfront surgery. Secondly, NECs have a salient response to chemotherapy. Finally, there is supposedly a decreased risk of distant treatment failure with induction chemotherapy in NECs [5]. After 3 cycles of Etoposide and Carboplatin, the resectability of the disease was confirmed on PET-CT imaging. The post operative histopathological features warranted adjuvant chemoradiation due to the presence of one lymph node in level IIA out of 49 total nodes dissected, measuring 1.6 cm, with pleomorphic nuclei, prominent nucleoli, positive for AE1/AE 3, EMA, INSM 1 on IHC studies indicating features of metastatic carcinoma of neuroendocrine differentiation. (Figures 1, 2 and 3)

In a nutshell, NECs presenting as MUO in the cervical lymph nodes are extremely rare with only two cases reported until date. These pathologies mandate appropriate diagnostic work up and a detailed IHC analyses to confirm the diagnosis. The possibility of neuroendocrine differentiation in a poorly differentiated or undifferentiated metastatic carcinoma of head and neck region should not be overlooked. Incorporating chemotherapy regimens as a part of the treatment plan either in the neo-adjuvant, adjuvant or definitive setting can yield promising results. Nevertheless, the rare occurrence and unusual disease presentation coupled with varied histological patterns and molecular aberrations discommode the establishment of standard treatment protocols for NECs of the head and neck region.
